# Overnight Social Isolation in Pigs Decreases Salivary Cortisol but Does Not Impair Spatial Learning and Memory or Performance in a Decision-Making Task

**DOI:** 10.3389/fvets.2015.00081

**Published:** 2016-01-11

**Authors:** F. Josef van der Staay, Annelieke J. Schoonderwoerd, Bo Stadhouders, Rebecca E. Nordquist

**Affiliations:** ^1^Emotion and Cognition Group, Department of Farm Animal Health, Faculty of Veterinary Medicine, Utrecht University, Utrecht, Netherlands; ^2^Brain Center Rudolf Magnus, Utrecht, Netherlands; ^3^Applied Biology, HAS University of Applied Sciences, Den Bosch, Netherlands

**Keywords:** isolation stress, cortisol spatial learning and memory, holeboard, working memory, reference memory, decision-making task, Iowa gambling task, pig (*Sus scrofa*)

## Abstract

Pigs in modern farming practice may be exposed to a number of stressors, including social stressors such as mixing or isolation. This may potentially affect both cognitive abilities and stress physiology of the animals. We tested the hypothesis that overnight social isolation in pigs impairs performance in a cognitive holeboard (HB) task (Experiment 1) and the Pig Gambling Task (PGT) (Experiment 2), a decision-making task inspired by the Iowa Gambling Task. In addition, we tested the effect of overnight social isolation on salivary cortisol levels. A within-subjects approach was used in which performance in the two behavioral tasks and cortisol levels were first determined during normal social housing, followed by performance and cortisol levels after experiencing stress induced by overnight social isolation. A total of 19 female pigs with a birth weight closest to their respective litter average was selected from 10 different litters and placed in two pens after weaning. Following habituation, pigs were trained in the HB task, starting at 10 weeks of age. Then, the pigs were isolated overnight, five individuals per night, at 15, 16, and 17 weeks of age. Between these three isolations, social housing and training in the HB continued. Starting 6 weeks after the end of the HB experiment, at approximately 23 weeks of age, the pigs were trained in the PGT. The effects of overnight social isolation on performance in this task were assessed once, when the pigs were 25 weeks old. Salivary cortisol was measured from samples collected 15 min after the start of isolation and at the end of the isolation period and compared to baseline values collected before the start of social isolation. Our results did not confirm the hypothesis that isolation impaired HB performance and decision-making in the PGT. Unexpectedly, overnight social isolation decreased cortisol levels below baseline values, an effect that was not associated with changes in performance of the behavioral tasks. We hypothesized that the housing and testing conditions may have prepared the animals to cope efficiently with stress.

## Introduction

In industrial pig production systems, animals are confronted with cognitive challenges. They must learn to recognize their penmates in order to establish and maintain a stable social hierarchy and must learn to use their pen environment optimally [see in Ref. (1, 2)]. In group housing systems, where automatic feeding stations are increasingly used, the pig must learn how to gain access to the food (3). Pigs are also exposed to various types of stressors, such as repeated regrouping (mixing), aggression of penmates, and unchallenging, barren environments. As social animals, pigs may experience stress if socially isolated from their penmates for a longer period of time (4, 5). Stress may interfere with or disrupt spatial memory performance and may compromise pigs’ cognitive abilities (2, 6) and affect their welfare (7).

Results of studies addressing the effects of adverse manipulations on cognitive performance of pigs are equivocal. There is some evidence for the notion that events such as confinement and isolation cause stress and interfere with subsequent cognitive performance [e.g., Ref. (2, 6)], whereas others found the effects of social isolation stress only in piglets younger than 35 days (8). Düpjan et al. (9), however, did not find effects of repeated isolation (a total of ten, 2½-h isolations during the course of seven successive days) on the performance of juvenile pigs in a cognitive bias task.

Pigs are able to discriminate between spatially distinct locations at a very young age, e.g., when developing a teat order. Teats can be considered as spatially distributed objects; once a specific teat preference is determined, the preference is preserved, even if the piglets are transferred to a foster sow (10). A broad range of tasks has been developed to test pigs’ cognition [reviewed by Ref. (11, 12)], such as spatial cognitive holeboard (HB) tasks (13–16), and more recently, a decision-making task (17). Pigs at weaning age and slightly older are already able to learn these tasks (13, 18, 19).

The spatial HB task allows measuring spatial working memory (WM) and reference memory (RM) simultaneously. In this task, food can only be found in a subset of potential sites (20–22). The WM “is a short-term memory that, once used, should be forgotten or ignored” [(23), p. 701] to avoid interference with the next trial. The WM holds information that is relevant only within a specific trial, such as a list of locations that have already been visited/explored during a particular trial. This measure represents the pig’s ability to avoid re-visits to baited holes during a trial (24).

The RM holds information about the solution of the spatial discrimination task, e.g., about the localization of the food, and that, once a food reward has been found and consumed, the hole will not be refilled during the trial. It also contains information about the actions necessary to get the bait (23, 25), for example, a head dip into a food-containing hole. This measure provides an index for the ability of pigs to discriminate between baited and unbaited holes (24). RM thus stores the general rules of a task, whereas the WM stores information that is relevant only within a specific trial (22).

The pig HB task has been used in a number of studies. All studies confirmed that Göttingen minipigs (16, 19, 26) and commercial pigs of different breeds were able to acquire this task [e.g,. Ref. (14, 15, 26–29)]. In Göttingen minipigs, performance in a HB task was slightly affected by a 9- and 38-day retention interval. After a 9-day retention interval, WM and RM performance was poorer than during the last trial block of the learning phase. Both WM and RM also decreased between the two memory phases (19).

In a study by Arts et al. (14) mixing, i.e., regrouping and housing of pigs with unfamiliar conspecifics, a practice that has been shown to induce stress, did not affect HB performance of well-trained pigs. Studies of HB performance of low birth weight (LBW) vs. normal birth weight (NBW) piglets have shown varying effects: either transiently reduced WM following reversal in LBW piglets (27) or improved RM performance in LBW compared to NBW piglets in both the acquisition and reversal phase of the HB task (13). In the latter study, the WM performance of the LBW was less disrupted than that of the NBW animals when switched to the reversal phase. Bolhuis et al. (15) and Grimberg-Henrici et al. (29) assessed the effects of environmental enrichment on HB performance in pigs. Bolhuis et al. (15) found that WM performance was better in enriched pigs than barren-housed piglets. In the study by Grimberg-Henrici et al. (29), the RM performance of the enriched-housed pigs was better than that of their barren-housed littermates during acquisition. During the reversal phase, enriched-housed pigs had a better general WM performance than the barren-housed pigs as indicated by reduced revisits to holes already visited during a trial, irrespective of whether they were of the baited or the unbaited set.

The HB task has also been used to assess the effects of dietary manipulations on cognitive performance in pigs. Haagensen et al. (16) tested the effects of a high fat and cholesterol, low carbohydrate diet or a low fat, high carbohydrate, and sucrose diet on the performance of Göttingen minipigs during the acquisition, after a retention interval, and during reversal learning in a HB. Both diets impaired WM and RM, compared to the standard diet, on retention and reversal. In a recent study assessing the effects of pre-weaning iron deficiency on post-weaning cognitive performance, Antonides et al. (18) showed a lasting impairment on the RM component during acquisition and reversal of the HB task.

For testing decision-making, a recently developed simple two-choice probabilistic task, the Pig Gambling Task (PGT) (17) was used. In this task, an advantageous option yields small but frequent rewards and a disadvantageous option yields large but infrequent rewards. In the long run, i.e., over a series of successive trials, choosing the advantageous option offers greater overall gain. This task is a modification of the Iowa Gambling Task (IGT) used to assess decision-making behavior under risk in humans (30). A main modification for use in animal research is the reduction of the number of “sets”: whereas human subjects can usually choose from four sets in the IGT, only two sets are presented in the animal modifications such as the PGT. This modification facilitates the performance of animals [e.g., rodents: (31); pigs: (17)] in the PGT.

Stress may affect decision-making behavior [reviewed by Ref. (32)]. For example, using the IGT, psychological studies with human subjects showed that judging and deciding are influenced by emotions and by the individual’s personality (33). Subjects exposed to stress made more unfavorable, disadvantageous choices, i.e., took more high-risk decisions, compared to unstressed subjects (34). In a study comparing the decision-making behavior of NBW piglets and LBW piglets in the PGT, the LBW piglets started to choose the advantageous option more often that the NBW piglets in the later phase of training (17). These piglets also performed the Judgment Bias Task in a manner that can be characterized as less optimistic than the NBW pigs. Murphy and colleagues (17) interpreted these findings as evidence that LBW pigs developed different behavioral strategies with respect to decision-making.

We addressed the question whether an adverse event, overnight social isolation, would interfere with performance in two different tasks on which pigs had been trained for a large number of training trials. We hypothesized that overnight social isolation would interfere negatively with spatial learning and memory performance in a HB task (i.e., that the pigs would make more WM and/or RM errors; Experiment 1), and with decision-making behavior in the PGT (i.e., that the pigs would make more disadvantageous choices; Experiment 2). We also expected that overnight social isolation would induce a physiological stress response, measured as increased salivary cortisol [e.g., Ref. (35)].

## Experiment 1: Spatial Holeboard Discrimination Task

### Materials and Methods

All methods of the two experiments were reviewed and approved by the local ethics committee (DEC Utrecht, **D**ier**E**xperimenten**C**ommissie) and were conducted in accordance with the recommendations of the EU directive 86/609/EEC. All efforts were taken to minimize the number of animals used and to avoid suffering.

#### Animals

Nineteen female piglets [Duroc × (Yorkshire × Finnish Landrace)] were selected at 4 weeks of age from 10 different litters. These piglets deviated <1 SD from the average birth weight of their litter. This criterion was used because we have seen in two previous studies that performance in LBW piglets differed from their normal weight littermates in the HB task [see in Ref. (13, 27)]. All piglets were born at the pig-breeding farm of University Utrecht, under conventional Dutch commercial pig housing conditions (tails were docked and plastic ear tags placed during the first week after birth).

Pairs of piglets from seven different litters (i.e., 14 piglets) fulfilled the above criterion. One piglet of a pair was randomly assigned to the first pen and the other to the second pen. Unfortunately, in the eighth litter, only one of the piglets was a female. This female was included in the study. In the ninth litter with more females, only one female fulfilled the selection criterion. In addition, from the tenth litter three female piglets close to the average litter weight were included in the study. Each of these five piglets was randomly assigned to one of the two pens. This procedure yielded 1 pen housing a group of 9, the other pen housing a group of 10 piglets. The 19 selected piglets were individually marked by ear tags and by spray-painted letters on the back to facilitate identification. The timeline of both experiments is summarized in Table 1.

**Table 1:** **Timeline of training and testing pigs in a holeboard task (Experiment 1) and the Pig Gambling Task (Experiment 2)**.

Age in weeks	Events
**Experiment 1: holeboard (HB) spatial orientation task**	
4	Nineteen piglets born at the farm of University Utrecht were weighed, selected, and transported to the nearby research stable. Random assignment of the piglets to pens (9 or 10 piglets per pen). Pigs were allowed to habituate to the new housing conditions and to new feed
5–7	Piglets were habituated to the experimenters and to consuming M&M’s^®^ that were used as reward in the behavioral tasks. The whole group was habituated to the hallway, waiting room, and testing room. Piglets were trained to chew voluntarily on cotton swabs for collection of saliva
8–10	Pigs were individually habituated to the hallway and testing room and the HB apparatus. During habituation sessions in the HB, bait was available in all 16 holes
10–14	Acquisition of the HB task during work days, with two trials per daily session. Four of the 16 holes contained bait. The acquisition phase lasted until the pigs had reached a reference memory score of 0.7, but at least 40 trials
14	Sampling of saliva on the last 3 days of the HB acquisition phase to determine baseline cortisol values
15–17	One overnight social isolation per week in three successive weeks. Four or five pigs were transferred to isolation pens from 15:00 to 9:00 the next morning. Saliva samples were taken 15 min after the isolation started (at 15:15) and at 9:00 the next morning
**Experiment 2: pig gambling task (PGT)**	
17	Pigs were moved to different pens in a part of the research stable near the PGT testing apparatus
21–22	Pigs were allowed to habituate to the new testing environment and equipment
23	Each pig was assigned to one of three successive test batches
23–24	Training of the three test batches started staggered on three successive days to enable testing the effects of overnight social isolation at the end of training at staggered time points.
	Training on the PGT for a total of 120 trials. Sampling of saliva on the first 3 days of the training phase of the PGT for determining baseline cortisol levels
25	One overnight social isolation. Saliva samples were collected for determining the effects of social isolation on cortisol levels; first sample in the afternoon, 15 min after isolation started (at 15:15), second sample immediately after the end of isolation, the next day at 9:00

#### Housing

Starting 1 day after weaning, the piglets were housed per group in two adjacent enriched pens (4 m × 5 m), situated in a naturally ventilated stable. Each pen contained a piglet nest, with rubber mats and straw bedding that could be accessed through transparent vertical plastic blinds. In addition, heat lamps ensured a comfortable temperature in the piglet nest. Bite sticks, balls, and gunny sacks provided additional enrichment. Both pens were cleaned daily and provided with fresh straw. Tap water was available *ad libitum* and piglets were fed twice a day. The ambient temperature during the study ranged from −3 to 22°C. Piglet health was monitored daily. During the first 5 days, piglets were allowed to acclimatize to the pens and to their penmates.

#### HB Apparatus

**Figure 1:**
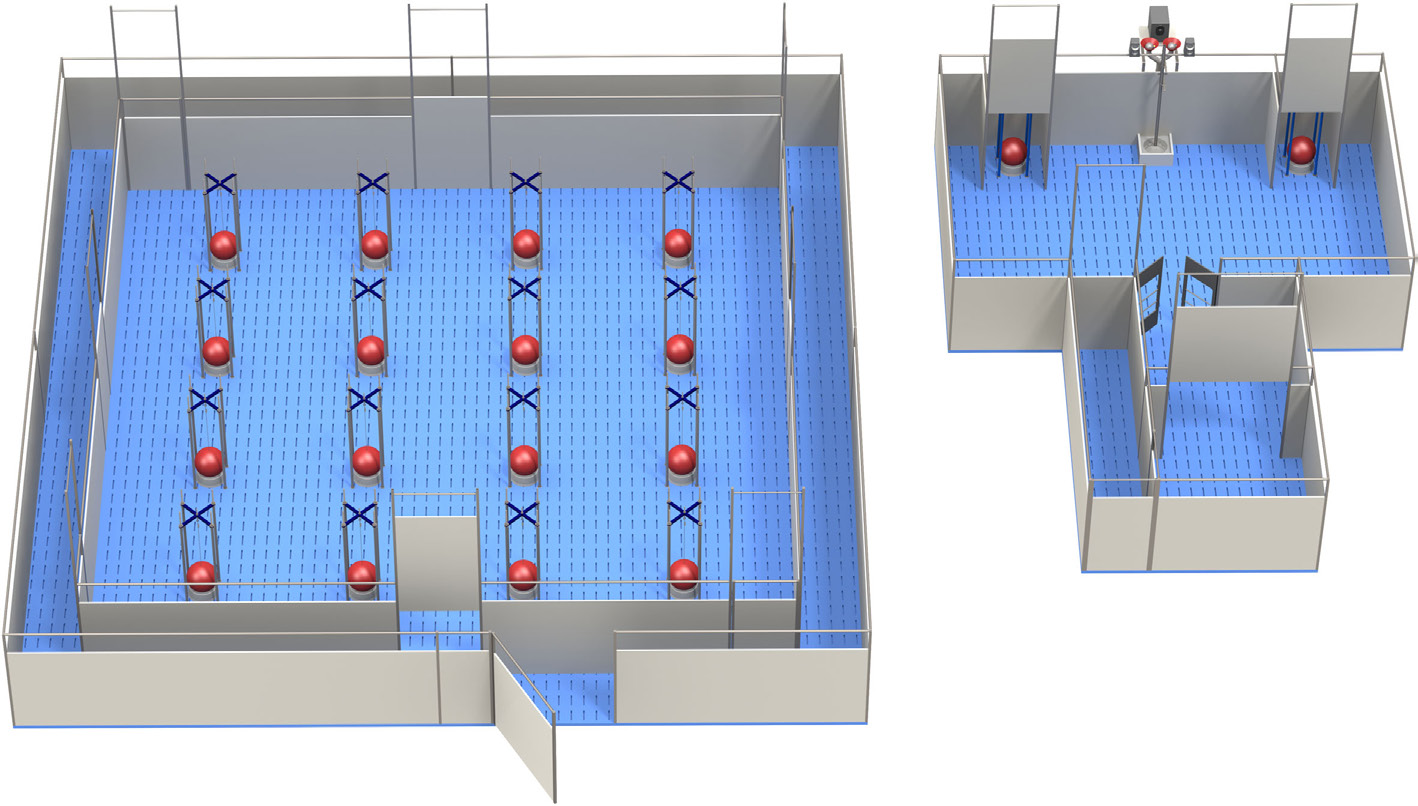
**The holeboard (left) and the apparatus for testing decision-making in pigs (the PGT: pig gambling task; right), side by side (Illustrations: Yorrit van der Staay)**.

The HB was a square arena (5.3 × 5.3 m), manufactured by Ossendrijver BV (Achterveld, The Netherlands), with a blue slatted floor and gray synthetic walls (80 cm height), with a steel bar on top of it (see Figure 1, left panel). The arena contained four guillotine doors, one at each side of the testing area, which could be opened by pulling a rope system from the outside of the test arena. The test area in the middle consisted of a 4 × 4 matrix with 16 food bowls. Beneath the food bowls there was a false bottom with four fresh M&M’s^®^ Milk Chocolates underneath, so the piglets could not search for the rewarded bowl by scent. Each bowl was covered by a ball (Jolly Ball Dog Toy, diameter: 24 cm, weight: 400 g) to prevent piglets from searching for rewards by sight [for details, see Fig. 1, panels 3 and 4 in Ref. (13)]. The experimental workflow was controlled, and hole visits were registered automatically using custom-made software (Bling Systems, Delft, The Netherlands). When a pig lifted a ball, this was scored as a hole visit; the signal was registered by an interface (LabJack) and sent to a computer [for details see in Ref. (13, 26)]. Lifting a ball was not counted as a (re)visit when it was lifted again within 10 s and no other holes were visited in between.

#### Isolation Pens

In a room adjacent to the pens housing the pigs, there were two rows with three isolation pens, each measuring 90 cm × 120 cm. The pens were separated by guillotine doors. Each pen measured 90 cm × 120 cm and was fitted with a drink nipple and a food bowl. The floor of the pens was covered with straw.

#### HB Habituation

During a habituation phase of 15 working days in three successive weeks, animals were allowed to explore the spatial HB in groups. During this phase, all holes were rewarded with chocolate M&M’s^®^. The piglets walked down the hallway to the waiting area (11.5 m^2^), next to the HB apparatus. The floor of the waiting area was covered with straw and water was available *ad libitum*. During this period, all piglets were trained to voluntarily approach the experimenter and chew on cotton swabs (in order to habituate them to the procedure of saliva sampling; see below).

On the first 3 days of the habituation phase, all piglets of a pen were led into the HB arena once daily, followed by three daily sessions with five piglets each, followed by 3 days with three or two piglets. Then, all pigs were habituated individually to the HB on three additional days: each pig was trained to walk around the test arena through a narrow, 40 cm wide corridor, until it found the opened guillotine door and entered the HB voluntarily (see Figure 1, left panel). All pigs were habituated two times a day, and stayed in the test area for 20 min. Per habituation trial, it was determined randomly which of the four doors gave access to the HB arena.

During HB habituation and training, the pigs were fed twice a day; they received ⅓ of the total amount of food before testing in the early morning and the remaining ⅔ after testing, late in the afternoon.

#### HB Acquisition

During the acquisition phase, starting 1 week after habituation, only four bowls contained bait. Every piglet had its own configuration of rewarded holes. Piglets received at least 20 days of testing with two trials a day in close succession until they had reached the criterion of a RM performance level of 0.7, averaged across two successive sessions (i.e., four trials). If the piglet did not reach this criterion, the training schedule was continued until the criterion was met (18, 28). This criterion ensured that all pigs had reached the same high performance level before we started to test the effects of overnight social isolation.

The testing area was cleaned daily with water, and the M&M’s^®^ underneath the false bottom of the food bowls [see in Ref. (13), Fig. 1, panels 3 and 4 for details] were replaced by new ones.

#### Testing the Effects of Overnight Social Isolation on HB Performance

After completion of the acquisition phase, training in the HB continued with daily sessions of two trials each on working days for 3 weeks. Once per week during this 3-week period, all pigs underwent overnight social isolation, for a total of three isolations per piglet. The order in which piglets were isolated was determined randomly. The 18-h isolation period started at 15:00 in the afternoon and lasted until 9:00 in the morning of the next day. Subjects stayed in a pen individually during one night, with *ad libitum* access to water. During the isolation period, piglets could hear and smell the pigs in the adjacent isolation pens, but could not see them.

#### Collection of Saliva

During the last 3 days of the acquisition phase in the HB apparatus, saliva from all subjects was collected for determining the baseline cortisol level according to Merlot et al. (35). As cortisol levels show a circadian rhythm, baselines were determined for the two timepoints that were later tested following isolation, i.e., at 9:00 in the morning and at approximately 15:15 in the afternoon of the same day. The pigs chewed on two cotton swabs (Cotton Swabs 150 mm × 4 mm WA 2PL; Heinz Herenz, Hamburg, Germany) until they were thoroughly moistened. The first sample during overnight social isolation was taken approximately 15–30 min after the isolation started at 15:00, i.e., at a time point where a peak in the cortisol response due to stress was expected [(5); e.g., Ref. (36, 37)], and on the next day at 9:00, at the end of isolation.

After saliva collection, the swabs were placed in special centrifuge tubes with inner cases (Salivette, Sarstedt, Germany) and were rapidly centrifuged (Sigma 4K10, supplier: Salm en Kipp bv, Breukelen, The Netherlands) at around 3524 g for 10 min at 10°C to obtain the saliva. The collected saliva was stored in the tubes at −20°C until cortisol concentration was measured (35) by a Coat-a-Count radioimmunoassay, according to manufacturer’s procedure (Coat-a-Count cortisol TKCO, Siemens Healthcare Diagnostics BV, The Hague, The Netherland). All samples from both experiments (HB and PGT) were assayed on the same day.

### Statistical Analysis

For the acquisition phase and for testing the effects of overnight social isolation in the HB task, two spatial memory components, WM and RM, and three latency/duration measures were analyzed [see also in Ref. (22)]: WM was calculated as number of rewarded visits divided by the number of visits to the baited set of holes (38). RM was calculated as number of visits to the baited set of holes divided by the number of visits to all holes (38). In addition, the latency of the first hole visit, the inter-visit interval [i.e., the time (s) between first and last hole visits divided through (number of hole visits – 1)], and the total trial duration (i.e., the time needed to find all food pellets, or the maximum trial duration, whatever event occurs first) were analyzed.

All analyses were performed using SAS version 9.4 (SAS Institute, Cary, NC, USA). The experimental unit was the individual pig because all effects tested are within-subjects behavioral changes or changes in cortisol levels. Normality of the untransformed variables was assessed by Shapiro–Wilk Test (SAS UNIVARIATE procedure). Variables measuring latencies or durations and the salivary cortisol data were log_10_-transformed to fulfill the normality requirement.

#### Acquisition of the HB Task

Means of blocks of four successive trials (i.e., two successive testing days) were calculated. All variables were subjected to an analysis of variance (ANOVA) with the within-subjects (repeated measures) factor Blocks of Trials (SAS GLM procedure).

#### Effects of Overnight Social Isolation on HB Performance

To assess the effects of overnight social isolation on HB performance, the means of the two trials of the session before isolation and the means of the two trials of the session after isolation were calculated and submitted to an ANOVA with the repeated measures factor Isolation (session before isolation vs. session following isolation), using the GLM procedure.

#### Effects of Overnight Social Isolation on Salivary Cortisol

The means of the three baseline afternoon samples of the baseline measurement and of the three baseline morning samples, and the means of the three isolation afternoon samples and of the three isolation morning samples were calculated and log_10_ transformed. Effects of isolation on salivary cortisol were analyzed using an ANOVA with the repeated measures factors Isolation (baseline vs. isolation) and Sampling time point (sampling at 15:15 vs. 9:00), using the SAS GLM procedure.

### Results HB

#### WM and RM During HB Acquisition

**Figure 2:**
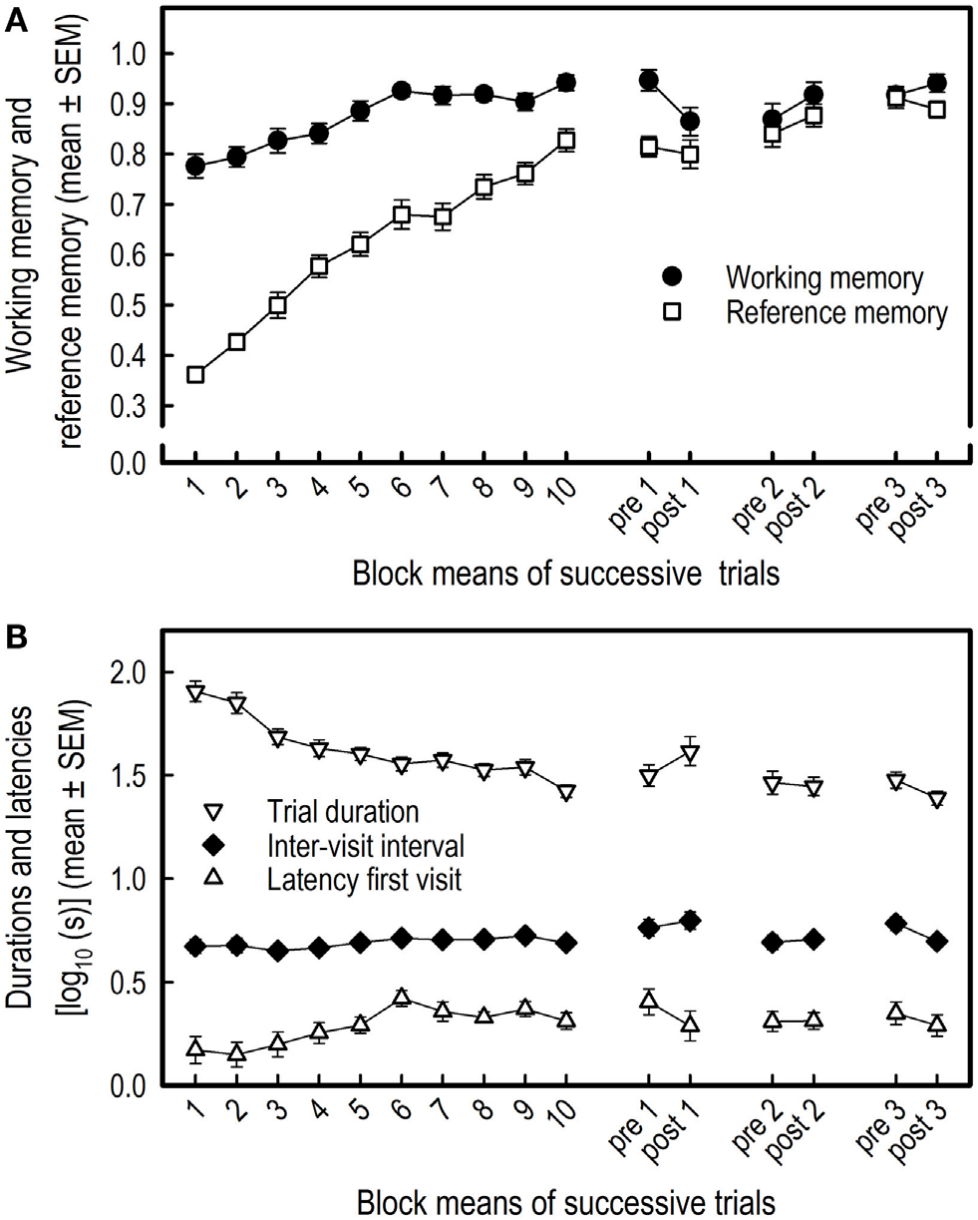
**Acquisition of the holeboard task and effects of three overnight isolations in 19 pigs**. The means and SEM of the working and reference memory performance **(A)** and of the log_10_ transformed latencies to first visit, inter-visit interval, and trial duration **(B)** are depicted. Blocks 1–10 represent 10 successive block means of four trials each, whereas pre 1, pre 2, and pre 3 represent block means of the two trials of the sessions before isolation, and post 1, post 2, post 3 represent the block means of the two trials of the sessions after isolation.

Both the WM performance (*F*_9,162_ = 11.07, *p* < 0.0001) and the RM performance (*F*_9,162_ = 61.72, *p* < 0.0001) increased during the course of training (Figure 2A).

#### Latencies and Durations

There was a slight increase in the mean latency of the first hole visit over time for all pigs (*F*_9,162_ = 4.10, *p* < 0.0001). Also, the inter-visit interval tended to increase over blocks (*F*_9,162_ = 1.67, *p* = 0.099) (Figure 2B). The total trial duration, however, decreased over the 10 successive blocks of trials (*F*_9,162_ = 20.10, *p* < 0.0001) (Figure 2B) due to the decrease of WM and RM errors.

#### Effects of Overnight Social Isolation on HB Performance

The isolation did not affect WM (*F*_1,18_ = 0.01, *p* = 0.944) nor RM of the pigs (*F*_1,18_ = 0.06, *p* = 0.815) (Figure 2A), and the isolation stress did not affect the latency to first hole visit (*F*_1,18_ = 1.51, *p* = 0.235), the inter-visit interval (*F*_1,18_ = 0.10, *p* = 0.7587), or the trial duration (*F*_1,18_ = 0.21, *p* = 0.654) (Figure 2B).

#### Effects of Overnight Social Isolation on Cortisol Levels

**Figure 3:**
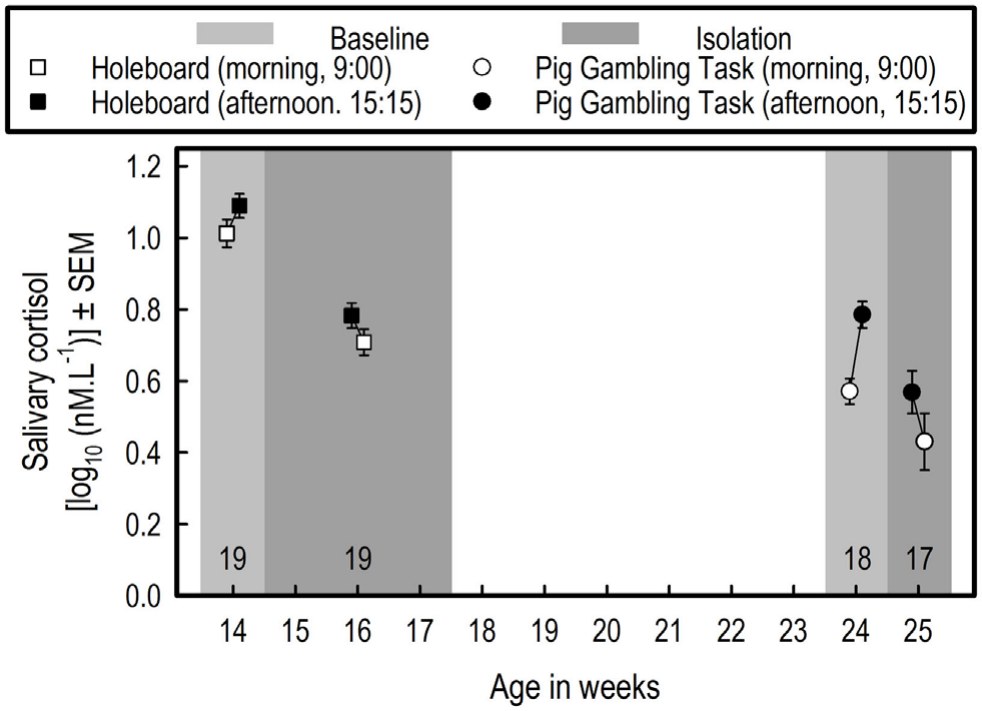
**Effects of overnight social isolation on salivary cortisol**. Saliva was collected on the last 3 days of the HB acquisition phase for determining baseline cortisol values. In three successive weeks (once per week), saliva was collected 15 min after the isolation started (at 15:15) and at 9:00 the next morning, for determining the effects of isolation on cortisol levels. For the HB experiment, the averages of the three baseline measurements and of the three measurements during isolations are depicted. For PGT, the averages of three baseline measurements are shown. Effects of social isolation were tested once in the PGT. Note that outliers detected by Grubbs’ test (http://graphpad.com/quickcalcs/grubbs1/) were excluded from statistical analysis.

Cortisol levels were lower during isolation than at baseline (*F*_1,18_ = 50.38, *p* < 0.0001), and in the morning, they were lower than in the afternoon (*F*_1,18_ = 6.76, *p* = 0.0181) (see Figure 3). There were no differential effects of isolation on this difference between the two sampling time points (afternoon–morning; Isolation by Sampling time point interaction: *F*_1,18_ = 1.47, *p* = 0.2411).

## Experiment 2: Decision-Making in the Pig Gambling Task

### Materials and Methods

#### Animals and Housing

The 19 pigs from the previous experiment were used. Approximately 3 weeks after the end of the HB experiment, the pigs were transferred to new pens (each measuring 4 m × 5 m) in another section of the same stable. The new pens were highly similar to those used during Experiment 1. The pigs were allowed to habituate to the new environment for 2 weeks. The testing apparatus was adjacent to the home pens.

#### Testing Equipment

The apparatus for testing pigs in the PGT consisted of a start box (1.2 m^2^), which was connected to a test chamber (3.6 m × 2.4 m) (see Figure 1, right panel). Access to the test chamber was controlled via a guillotine door, remotely operated by an experimenter. The left and right corner of the testing arena contained a “goal box” each (0.4 m wide) containing a bowl, which was covered by a red hard-plastic large ball. The pigs were trained to perform the operant response of lifting the ball that covered the food bowl. If the response was rewarded, the appropriate number of M&M’s^®^ was delivered into the central food bowl between the two goal boxes [for technical details, see in Ref. (17), Fig. 1B–D].

#### Habituation and Training in the PGT

Because they were already habituated to the experimenters and to performing behavioral tasks alone, the pigs were only habituated 1 day in the PGT apparatus. The pigs were allowed to explore the test setup with M&M’s^®^ underneath the balls in the goal boxes and in the central food bowl. Then, the pigs were trained individually to lift the ball in one goal box, whereas the other goal box stayed closed. Both sides were open equally often to prevent that the pigs developed a side preference. After pushing up the ball, an M&M’s^®^ reward was made available in the central food bowl (see Figure 1, right panel). When the pigs reliably showed the required response, the next phase started.

At this point, each pig was assigned to one of three test batches, and training of the three test batches started staggered on three successive days to enable testing the effects of overnight social isolation at the end of training at staggered time points.

At the start of each training trial, the guillotine doors of both goal boxes were open. As soon as the pig had lifted the ball in one of the goal boxes, the other box was closed (to prevent the pig from choosing the other goal box). The appropriate number of M&M’s^®^ fell through a vertical tube into the central food bowl that was covered by a transparent lid. If the animal selected the disadvantageous side, four M&M’s^®^ were released into the food bowl [see in Ref. (17), Fig. 1C] and the reward was made accessible in 3 trials of a series of 10 trials by rising the lid that covered the food bowl [see in Ref. (17), Fig. 1D]. Two M&M’s^®^ were released into the food bowl when the pig chose the advantageous side, and the reward was made accessible in 8 trials of a series of 10 trials.

**Figure 4:**
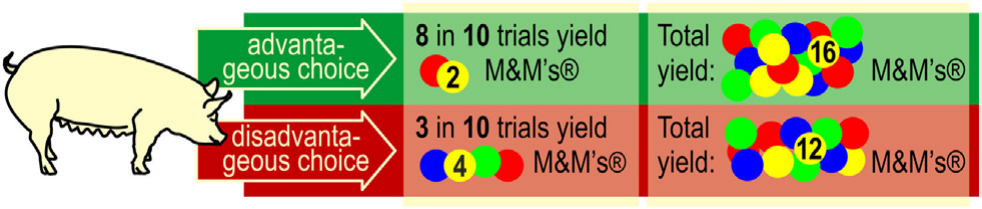
**In the decision-making task, a pig can choose between two sides, an advantageous, and a disadvantageous side (see also the PGT apparatus; Figure 1, right panel)**. Pigs should learn to respond to the advantageous side because in the long run, this choice yields the largest number of rewards (M&M’s^®^).

Over 12 daily sessions with 10 trials each (total: 120 trials), pigs could choose freely between the two goal boxes. Any correct response in a goal box resulted in the delivery of reward into the central food bowl. The number of M&M’s^®^ and the accessibility of reward were predetermined. A response in the advantageous goal box yielded a small quantity of reward (two M&M’s^®^) but had a high probability (80%) that the rewards would be made accessible. A response in the disadvantageous goal box yielded higher quantities of reward (four M&M’s^®^), but there was a low probability (30%) that the rewards were made accessible. In each series of 10 successive trials, the advantageous option yielded 16 accessible M&M’s^®^ while the disadvantageous option yielded 12 accessible M&M’s^®^ (see Figure 4).

In rewarded trials, as soon as the pig moved to the central food bowl, the lid was raised giving access to the M&M’s^®^. In unrewarded trials, rewards were also delivered after the pig had pushed up the ball in the goal box, but the lid stayed closed and the reward remained inaccessible (which may be experienced as “punishment” by the pigs). After each trial, pigs were allowed to return to the start box for the next trial 25 s after making a choice. The order in which the rewards were accessible and inaccessible differed daily but the probability of getting a reward remained the same within each series of 10 trials. The number of advantageous choices was recorded per pig for each of the six blocks of 20 trials.

#### Testing the Effects of Overnight Social Isolation on the Percent Advantageous Choices

After the 12th training day, the pigs were socially isolated overnight (from 15:00 to 9:00), using the isolation pens from the first experiment. In the morning, after isolation on day 13, the pigs were tested again. The pigs received 10 trials per session. The number of advantageous and disadvantageous choices was registered and expressed as percentage advantageous choices for each of these sessions. We calculated and analyzed the percentage of advantageous choices for comparability reasons: we considered sessions of 10 or 20 trials in the statistical analyses.

#### Collection of Saliva

During the first week of training on the PGT, saliva from all subjects was collected by letting the pigs chew on cotton swabs during 5 min twice, at 9:00 in the morning and at approximately 15:15 in the afternoon, two subjects at a time, to determine the baseline cortisol level according to Merlot et al. (35). Saliva was collected on three consecutive days. On the last (12th) training day in the PGT in the afternoon, the first salivary sample was collected, and on the next morning, the second saliva sample was collected. Then, the pigs were tested in the PGT for the last time (13th training day).

### Statistical Analysis

One pig had to be excluded from analyses because it refused to enter the test arena. After checking whether the variables were normally distributed using the Shapiro–Wilk test (UNIVARIATE procedure), they were analyzed by a repeated measures ANOVA with the (within-subjects) factor Blocks.

**Figure 5:**
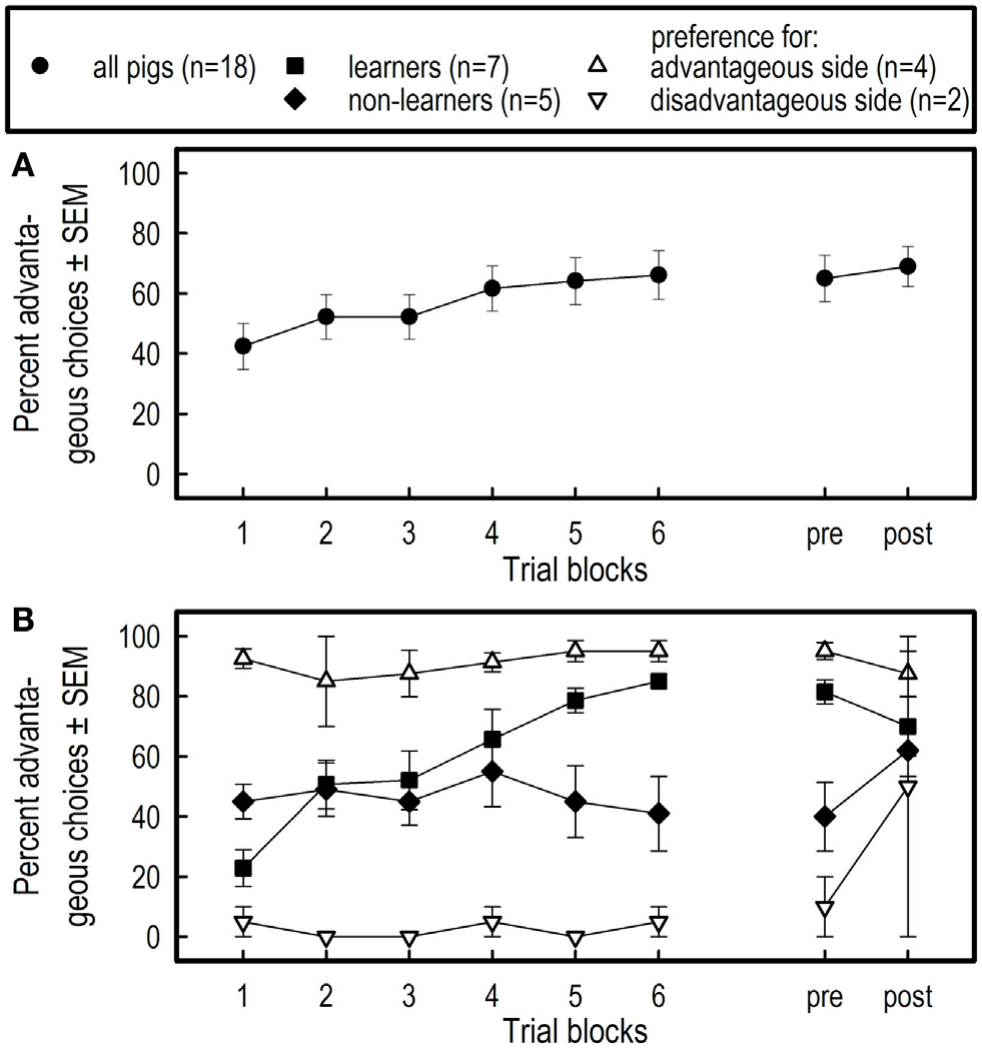
**Mean percentage (±SEM) of advantageous choices per block of 20 trials and during the session of 10 trials pre- and the session of 10 trials post-isolation across all pigs (A), and across pigs qualified as learners, non-learners, preferring the advantageous or disadvantageous side (B)**. Note that only two pigs preferred the disadvantageous side, from which one switched to the advantageous side after overnight social isolation.

Inspection of the individual learning curves revealed that some pigs learned the task, i.e., increased the number of advantageous choices across blocks, some pigs did not learn the task (no change of advantaged choices across blocks, but also no obvious side preferences), whereas others showed persistent side preference as seen in Figure 5B. The pigs were classified *a posteriori* as learners (*N* = 7), non-learners (*N* = 5), advantageous side preferring (*N* = 4), or disadvantageous side preferring (*N* = 2). Advantageous side preferring was defined as 17–20 advantageous choices, whereas disadvantageous side preferring was defined as 17–20 disadvantageous choices per block of 20 trials in at least four of the six blocks of the learning phase. Differences in learning between the four subgroups defined *a posteriori* were analyzed by ANOVA with the repeated measures factor Blocks and between subjects factor Subgroups.

#### Effects of Overnight Social Isolation on Choices in the PGT

To assess the effects of overnight social isolation on HB performance, the percent advantageous choices in the last 10 trials of the session before isolation and in the 10 trials of the session after isolation were calculated and submitted to a repeated measures ANOVA with the factor Isolation (session before isolation vs. session following isolation), using the GLM procedure.

#### Effects of Isolation Between Subgroups

Effects of isolation between subgroups were analyzed by ANOVA with the between subjects factor Subgroups (learners, non-learners, advantageous side preferring, disadvantageous side preferring) and the repeated measures factor Isolation (session 12, before isolation vs. session 13, after isolation).

#### Comparison of Cortisol Baseline Levels of the Two Experiments

We compared the baseline cortisol levels measure in the two experiments by an ANOVA with the repeated measures factors Experiment (mean baseline cortisol of first vs. mean baseline cortisol of second experiment) and Sampling time point (sampling at 15:15 vs. 9:00), using the GLM procedure.

#### Effects of Overnight Isolation on Salivary Cortisol

The means of the three baseline afternoon samples and the three baseline morning samples were both calculated and log_10_ transformed. Effects of isolation on salivary cortisol were analyzed using an ANOVA with the repeated measures factors Isolation (mean baseline cortisol vs. cortisol during isolation) and Sampling time point (sampling at 15:15 vs. 9:00), using the GLM procedure.

### Results PGT

#### All Pigs

Averaged over all pigs, the percentage of advantageous choices increased across the six successive blocks (*F*_5,85_ = 4.53, *p* = 0.001) (see Figure 5A). The overnight social isolation had no effect on the pigs’ choices (comparison of performance on day 12 and day 13; *F*_1,17_ = 0.36, *p* = 0.5547).

#### Four a Posteriori Subgroups

Averaged over the six successive blocks of the training phase, the four subgroups differed for the number of advantageous choices (Subgroups; *F*_3,14_ = 23.49, *p* < 0.0001) (see Figure 5B). A marginal Block effect (*F*_5,70_ = 2.32, *p* = 0.0524) and a block by subgroups interaction effect (*F*_15,70_ = 3.20, *p* = 0.0005) indicate that the learning curves of the four groups were indeed different.

As a consequence of large differences in learning between the four subgroups, the average percent advantageous choices across the 12th (before isolation) and 13th day (after isolation) was different between subgroups (*F*_3,14_ = 6.70, *p* = 0.0050). Overnight social isolation did not affect the percent choices between subgroups on day 12, compared with day 13 (Isolation: *F*_1,14_ = 2.32, *p* = 0.1499; Isolation by Subgroups interaction: *F*_3,14_ = 2.92, *p* = 0.0711).

#### Comparison of the Baseline Cortisol Measurements

On average, the cortisol baseline values in the HB experiment (Experiment 1) were higher than those in the PGT experiment (Experiment 2) (*F*_1,17_ = 121.41, *p* < 0.0001) (see Figure 3). In both experiments, the levels measured at 9:00 were lower than those measured at 15:15, but the difference was larger in the PGT experiment (sampling time point, *F*_1,17_ = 42.04, *p* < 0.0001; Experiment by Sampling time point interaction, *F*_1,17_ = 10.61, *p* = 0.0046).

#### Effects of Overnight Social Isolation on Cortisol Levels

The data of 17 pigs were used, due to exclusion of one pig with an outlier cortisol morning measurement (see Figure 3). Cortisol levels were lower during isolation than at baseline (*F*_1,16_ = 8.13, *p* = 0.0115), and in the morning, they were lower than in the afternoon (*F*_1,16_ = 6.76, *p* = 0.0181). There was no differential effect of isolation on this difference between the two sampling time points (i.e., afternoon – morning; Isolation by Sampling time point interaction: *F*_1,16_ = 0.36, *p* = 0.5547).

## Discussion

Stress may affect cognitive functioning and decision-making behavior [reviewed by Ref. (32)]. The present study investigated the effects of overnight social isolation in pigs on performance in a spatial HB task, in the PGT, and on salivary cortisol as physiological stress marker. We did not observe effects of overnight social isolation on either behavioral task. This lack of effect was already evident after the first overnight social isolation, and consequently, it cannot be ascribed to a habituation effect of repeated isolations. This result contrasts with findings by Laughlin et al. (6). Overnight social isolation did affect cortisol levels. However, contrary to our hypothesis, we observed decreases in cortisol levels when measured both at 15 min following the start of social isolation and after 18 h of social isolation. This decrease was observed at both periods in which social isolation was performed, at 15−17 weeks and at 25 weeks of age.

### Spatial Learning and Memory in the HB

The pigs learned the HB task readily, corroborating earlier findings [e.g., Ref. (13, 14, 27)]. All piglets were trained to a RM criterion of 0.7 – this ratio measure reflects the ability of the animals to avoid re-visits to baited holes during a trial and reaches the value 1.0 in error-free trials (22). Then, they were exposed to the overnight social isolation. However, overnight social isolation did not affect the spatial WM or RM performance in the HB task, nor did it affect latencies or durations in this task. Our results are in line with a study by Arts et al. (14), in which no differences were found in pig HB performance following a social stressor, namely regrouping of pigs (mixing). Latency and duration measures can be taken to reflect pigs’ motivation. The lack of change in performance after isolation thus indicates that the isolation did not affect pigs’ motivation to perform the task or work for rewards (22, 24). We may conclude that neither stress induced by overnight social isolation nor by mixing affected subsequent HB performance. It is also possible that the pigs did not find overnight social isolation stressful, as discussed below in relation to the cortisol measures.

### Decision-Making Behavior in the PGT

The learning curve in the PGT based on the performance averaged over all pigs (see Figure 5A), suggests that the pigs had learned the task, albeit to a moderate performance level. The level reached is comparable to that reported by Murphy et al. (17) who studied decision-making behavior in LBW and NBW piglets. However, as depicted in Figure 5B, not all pigs learned the task and increased choices of the advantageous side in the present study. Thus, the learning curve calculated across all pigs is not a relevant representation of the pigs’ behavior. Based on their choice behavior during the entire training period of 120 trials, only 7 of the 18 pigs were classified *a posteriori* as learners; they increased the number of advantageous choices during training. Five pigs showed no learning at all, i.e., they randomly chose the advantageous or disadvantageous side. This group was classified as non-learners. Four of the 18 pigs showed directional persistence and were classified as preferring the advantageous side and two pigs were classified as preferring the disadvantageous side, i.e., the latter two groups showed a strong side preference or side bias. Persistent side preference is difficult to break in pigs [e.g., Ref. (39, 40)]. Side bias prevents learning about the contingencies that are in effect at the other side (goal box). Overnight social isolation did not affect the pigs’ choice behavior in any of the four subgroups.

**Figure 6:**
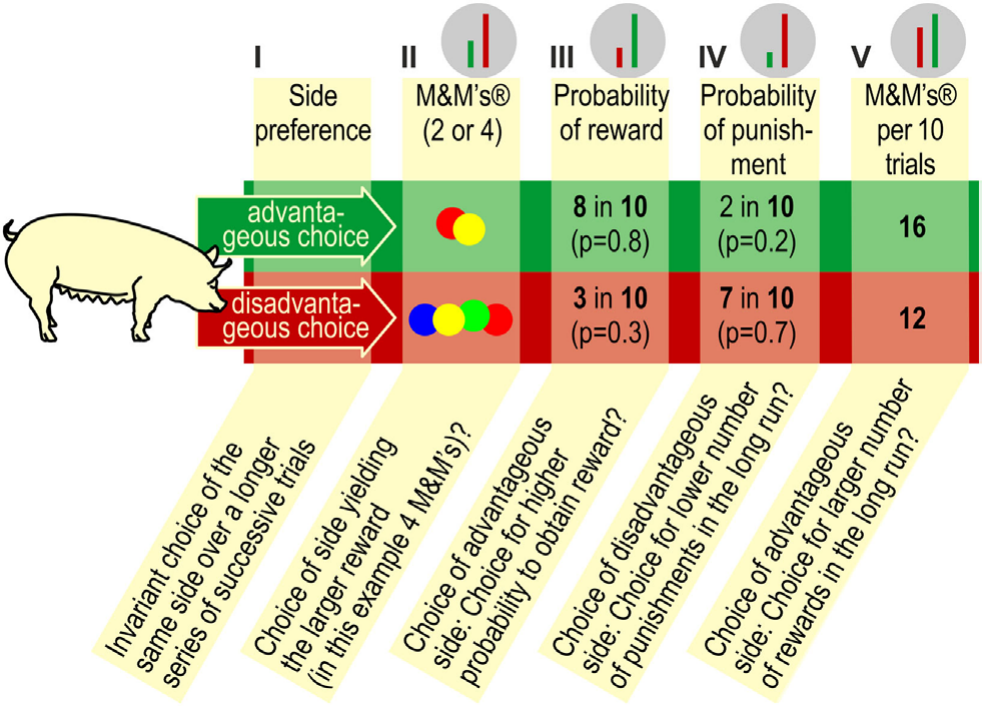
**Contingencies in the PGT**. Pigs may choose (I) invariably either the left or right goal box (side preference, side bias; as long as the pig persists in selecting one side, it will not learn anything about the contingencies that are in effect on the other side), (II) the side that yields the larger reward (i.e., four M&M’s^®^), (III) the side that yields reward with the highest probability in the long run, (IV) the side, which yields the lowest probability of punishment, i.e., non-reward in the long run, or, finally, (V) the side, which renders the largest number of M&M’s^®^ in the long run. The green and red bars against the gray background on top of the figure show the relative contrast between the advantageous and disadvantageous choices, depending on the contingency according to which the pig chooses. It is obvious that the contrast between the advantageous and disadvantageous choices is lowest with option (V).

As described in the Section “introduction”, gambling tasks in animal research use a reduced number of “sets” to choose from compared to human gambling tasks. This modification makes the tasks easier for animals to learn but also reduces the number of choice alternatives. A small number of discriminable contingencies and consequences underlies the advantageous and disadvantageous choices (see Figure 6). Given the increased usage of gambling tasks in animal research and the variability we have observed in performance of the task, it is worthwhile to consider the potential interpretations of performance in these types of tasks.

It remains to be demonstrated which contingencies animals detect and which ones guide their choice behavior. Moreover, Anselme (41) raised doubt about the notion that this type of tasks models decision-making under risk. He argues that “(….) *opportunity costs are only a source of risk provided that they imperil (in part or in totality) an individual’s own limited resources*” [(41), p. 120]. Failing to gain the maximum of 16 M&M’s^®^ per series of 10 choices has no serious consequences. Because, contingent on lifting a ball in a goal box, M&M’s^®^ were released into the central food bowl, the sound of the falling M&M’s^®^ acted as a secondary reinforcer that helped to maintain responding. Consuming accessible M&M’s^®^ served as primary reinforcer, whereas leaving the M&M’s^®^ inaccessible after hearing the sound of M&M’s^®^ falling into the food trough may have been perceived as mild punishment.

When responding in the gambling task, pigs may choose (I) the preferred side (side preference, side bias), (II) the side yielding the larger reward (more M&M’s^®^), (III) the side that yields reward with the highest probability, (IV) the side that yields the fewest punishments, i.e., impedes consumption of the M&M’s^®^ in the central food bowl, or, finally, (V) pigs may choose the side that, within series of 10 trials, yields the largest number of M&M’s^®^ (see Figure 6). It has not been established whether pigs are able to assess the probabilities of earning maximum number of reward/minimum number of punishment in the long run. It is conceivable, and perhaps more likely, that they simply choose the contingency that provides reward in the majority of trials, where the number of chocolate M&M’s^®^ is not relevant. Based on the set of contingencies in effect in the present study, it is not possible to distinguish between probabilities III, IV, and V.

In an earlier study, we found that pigs needed a large number of trials to acquire successive and conditional learning tasks (42). In the present study, only seven pigs were able to detect the contingencies that yielded the maximum reward. It remains to be determined whether this modification of the task is suited for testing pigs, and how many trials are needed to train the pigs to consistently make advantageous choices.

One could argue that the lack of effect of social isolation on behavior in the two experiments is due to habituation to the isolation procedure. This explanation, however, is unlikely. First, even the very first isolation during the HB task did not affect cognitive performance in the session following isolation. Second, the pigs did not experience any social isolation in the period of approximately 1½ months between the HB task (Experiment 1) and PGT (Experiment 2). Because we expected to find robust effects of social isolation stress on performance in subsequent behavioral tests, and to keep the number of animals used to a minimum, we decided to use the same animals in both experiments.

### Salivary Cortisol

#### Age-Associated Decrease of Baseline Cortisol

Baseline salivary cortisol measures at 19 weeks of age were considerably lower than at 14 weeks of age. This observation corroborates earlier findings by de Jong et al. (43) who reported a steep drop of salivary cortisol in pigs from the age of 15–22 weeks. However, Hillmann et al. (44) reported that the cortisol level increased with age and body mass of pigs.

#### Circadian Fluctuation of Cortisol

Cortisol levels show a circadian rhythm. A general finding is that cortisol levels are lower during the night than during daytime (43, 45, 46). We expected, based on these publications, that the baseline cortisol levels at 9:00 and 15:15 should be similar. According to Hillmann et al. (44), the circadian pattern of cortisol is more pronounced with increasing age in pigs.

Provided that cortisol levels of a treatment are compared with a pre-treatment baseline, salivary cortisol can provide a good indication of the HPA response to a stressor (35). As sampling of saliva is considered stress-free (47), it is unlikely that this procedure affected the stress state of the pigs (48), all the more because the pigs had been thoroughly trained to chew voluntarily on the cotton swabs.

#### Effects of Social Isolation on Salivary Cortisol Levels

In the present study, the cortisol level during isolation(s) was lower than that of the baseline measurements. Considering that the baseline measurements in the first and second experiment were separated by a 10-week time period (see Table 1; Figure 3), in which the baseline levels dropped considerably [corroborating the results of de Jong et al. (43), but not of Hillmann et al. (44)], one could hypothesize that the effects of isolation simply reflect the effects of aging. However, the period between baseline and isolation measurements was only 1 week for the first isolation in Experiment 1, and for the isolation in Experiment 2. An additional statistical comparison between baseline and first isolation in Experiment 1 (data not shown) makes this hypothesis unlikely. Therefore, we assume that the drop in cortisol levels in isolation sessions does not merely reflect effects of an age-related drop in cortisol.

The decrease in cortisol that we observed following exposure to social isolation in both tests was an unexpected finding. We assume that social isolation is a stressor, and cortisol levels generally increase following acute exposure to a stressor. Certainly our first measures, 15 min after being placed in social isolation, would be expected to reflect an acute response to the situation. Interestingly, three separate studies have shown that individually housed gilts had decreased salivary cortisol levels compared to gilts housed in groups (49–51), and that group-housed gilts showed a decrease in cortisol after transfer to individual housing in farrowing crates (50). Based on these and the present study, group housing with all of the social interactions that it entails may provide more stress than social isolation as operationalized in the present study.

The decrease in cortisol observed in the animals following social isolation may be related to the housing as it was operationalized in the present study. One hypothesis, as put forward by Geverink et al. (49), is that the decrease in cortisol may be an effect of decreased activity levels in individually housed animals, as activity and exercise are known to increase cortisol levels. The animals were not restrained during social isolation, but had much less space than during group housing and were not stimulated by conspecifics to move around in the pen. Furthermore, the isolation pens were situated next to each other. The pigs in these pens had *ad libitum* access to water and food, and could root in the straw that covered the floor.

All pigs could hear and smell their isolated neighbors. Notably, “social support might not require all senses but rather rely on a few senses that are important to that particular species” [(52), p. 7]. Social support can dampen stress reactivity (53), and can help animals to cope with stressful events (52). Social support most likely occurs in stable social groups like the ones in the present study. Familiarity and a social bond are considered as minimum requirements for social support to occur (52). Both the provider and the receiver of social support may benefit, i.e., stress reactivity in the isolation condition may have been dampened in all pigs, albeit through different mechanisms (53).

Low and high stress levels seem to impair cognitive performance, whereas intermediate stress levels may even facilitate learning and memory (54). In the HB task, the pigs had already reached a high level of performance before they were subjected to social isolation. Consequently, it is unlikely that the (mild) stress that they experienced during social isolation improved performance post isolation. In the PGT, comparison of the subgroups, and separate analysis of the “learners” did not reveal any differential effect of social isolation. If the training itself is not very stressful, or if stress is experienced long after training, its consequences on cognitive performance are less predictable (55). In order to keep the behavioral training itself stress free, we extensively habituated the pigs to all aspects of the testing procedures and testing environment.

Our training and testing procedure itself may be considered as “cognitive enrichment” [see also in Ref. (29)]. This type of enrichment seems to be able to reduce excitement and fear in pigs (56) and may thus be able to dampen stress responses. For example, Zebunke et al. (57) trained pigs to approach a “call feeding station” approximately 30 times a day and to operate a button on a fixed ratio schedule to earn a portion of feed. They concluded that the cognitive enrichment can reduce stress, as measured by heart rate and heart rate variability. In line with this study, Siegford et al. (58) reported that training in a spatial maze task reduced the stress response of 12-day-old piglets and reduced their fear response, measured at 7 weeks of age, i.e., that maze training may reduce fear of novel persons and ameliorate cognitive deficits. Their study suggests that the exposure of young male piglets to environments that requiring spatial learning is beneficial, an effect not found in females. The enrichment effects of training and testing *per se* may thus interfere with the aim of the present study to demonstrate an adverse effect of the stress induced by overnight social isolation.

## Conclusion

Overnight social isolation did not affect pigs’ behavior in a HB task and had no effect on pigs’ decision-making in the PGT, in line with observations by Düpjan et al. (9) and Murphy et al. (59). However, the average cortisol level during isolation was lower than the average cortisol level during baseline, indicating that the isolation affected physiological but not behavioral measures. During overnight social isolation, the pigs could still smell and hear their penmates. They had straw as bedding and could move around. These conditions might have reduced the averseness of the overnight social isolation to a level that effects on performance during behavioral testing were not detectable, whereas the salivary cortisol even decreased during social isolation.

## Author Contributions

FS and RN designed the study and wrote the final version of the manuscript. AS and BS performed the study, collected the data, and wrote the two BSc theses on which this manuscript is based. FS performed the data analysis. All authors approved the final manuscript.

## Conflict of Interest Statement

The authors declare that the research was conducted in the absence of any commercial or financial relationships that could be construed as a potential conflict of interest.

## References

[1] Held S Mendl M Laughlin K Byrne RW Cognition studies with pigs: livestock cognition and its implication for production. J Anim Sci (2002) 80:E10–7. 10.2134/animalsci2002.0021881200800ES10003x

[2] Mendl M Laughlin K Hitchcock D. Pigs in space: spatial memory and its susceptibility to interference. Anim Behav (1997) 54:1491–508. 10.1006/anbe.1997.0564 9794774

[3] Ernst K Puppe B Schön PC Manteuffel G A complex automatic feeding system for pigs aimed to induce successful behavioural coping by cognitive adaptation. Appl Anim Behav Sci (2005) 91:205–18. 10.1016/j.applanim.2004.10.010

[4] Herskin MS Jensen HK Effects of different degrees of social isolation on the behaviour of weaned piglets kept for experimental purposes. Anim Welf (2000) 9:237–49.

[5] Ruis MAW Te Brake JHA Engel B Buist WG Blokhuis HJ Koolhaas JM Adaptation to social isolation – acute and long-term stress responses of growing gilts with different coping characteristics. Physiol Behav (2001) 73:541–51. 10.1016/S0031-9384(01)00548-0 11495658

[6] Laughlin K Huck M Mendl M Disturbance effects of environmental stimuli on pig spatial memory. Appl Anim Behav Sci (1999) 64:169–80. 10.1016/S0168-1591(99)00036-2

[7] Ohl F van der Staay FJ. Animal welfare: at the interface between science and society. Vet J (2012) 192:13–9. 10.1016/j.tvjl.2011.05.019 21703888

[8] Kanitz E Hameister T Tuchscherer M Tuchscherer A Puppe B. Social support attenuates the adverse consequences of social deprivation stress in domestic piglets. Horm Behav (2014) 65:203–10. 10.1016/j.yhbeh.2014.01.007 24486118

[9] Düpjan S Ramp C Kanitz E Tuchscherer A Puppe B A design for studies on cognitive bias in the domestic pig. J Vet Behav Clin Appl Res (2013) 8:485–9. 10.1016/j.jveb.2013.05.007

[10] Skok J Škorjanc D A note on precise tracking of suckling position by piglets. Arch Für Tierz (2014) 57:1–7. 10.7482/0003-9438-57-011

[11] Gieling ET Nordquist RE van der Staay FJ Assessing learning and memory in pigs. Anim Cogn (2011) 14:151–73. 10.1007/s10071-010-0364-3 21203792 PMC3040303

[12] Kornum BR Knudsen GM. Cognitive testing of pigs (*Sus scrofa*) in translational biobehavioral research. Neurosci Biobehav Rev (2011) 35:437–51. 10.1016/j.neubiorev.2010.05.004 20553757

[13] Antonides A Schoonderwoerd AC Nordquist RE van der Staay FJ Very low birth weight piglets show improved cognitive performance in the spatial cognitive holeboard task. Front Behav Neurosci (2015) 9:10 10.3389/fnbeh.2015.00043 25774127 PMC4343021

[14] Arts JWM van der Staay FJ Ekkel ED. Working and reference memory of pigs in the spatial holeboard discrimination task. Behav Brain Res (2009) 205:303–6. 10.1016/j.bbr.2009.06.014 19539660

[15] Bolhuis JE Oostindjer M Hoeks CWF de Haas EN Bartels AC Ooms M Working and reference memory of pigs (*Sus scrofa* domesticus) in a holeboard spatial discrimination task: the influence of environmental enrichment. Anim Cogn (2013) 16:845–50. 10.1007/s10071-013-0646-7 23740471

[16] Haagensen AMJ Klein AB Ettrup A Matthews LR Sørensen DB Cognitive performance of Göttingen minipigs is affected by diet in a spatial hole-board discrimination test. PLoS One (2013) 8:e79429 10.1371/journal.pone.0079429 24223947 PMC3818226

[17] Murphy E Kraak L van den Broek J Nordquist R van der Staay FJ. Decision-making under risk and ambiguity in low-birth-weight pigs. Anim Cogn (2015) 18:561–72. 10.1007/s10071-014-0825-1 25527296

[18] Antonides A Schoonderwoerd AC Scholz G Berg BM Nordquist RE van der Staay FJ Pre-weaning dietary iron deficiency impairs spatial learning and memory in the cognitive holeboard task in piglets. Front Behav Neurosci (2015) 9:16 10.3389/fnbeh.2015.00291 26578919 PMC4626557

[19] Haagensen AMJ Grand N Klastrup S Skytte C Sørensen DB Spatial discrimination and visual discrimination: two methods evaluating learning and memory in juvenile Göttingen minipigs. Behav Pharmacol (2013) 24:172–9. 10.1097/FBP.0b013e32836104fd 23542905

[20] Olton DS Becker JT Handelmann GE Hippocampus, space, and memory. Behav Brain Sci (1979) 2:313–65. 10.1017/S0140525X00062713

[21] Olton DS Samuelson RJ Remembrance of places passed: spatial memory in rats. J Exp Psychol Anim Behav Process (1976) 2:97–116. 10.1037/0097-7403.2.2.97

[22] van der Staay FJ Gieling ET Espitia Pinzón N Nordquist RE Ohl F The appetitively motivated “cognitive” holeboard: a family of complex spatial discrimination tasks for assessing learning and memory. Neurosci Biobehav Rev (2012) 36:379–403. 10.1016/j.neubiorev.2011.07.008 21810442

[23] Dudchenko PA. An overview of the tasks used to test working memory in rodents. Neurosci Biobehav Rev (2004) 28:699–709. 10.1016/j.neubiorev.2004.09.002 15555679

[24] Bouger PC van der Staay FJ. Rats with scopolamine- or MK-801-induced spatial discrimination deficits in the cone field task: animal models for impaired spatial orientation performance. Eur Neuropsychopharmacol (2005) 15:331–46. 10.1016/j.euroneuro.2004.11.006 15820423

[25] Frick KM Baxter MG Markowska AL Olton DS Price DL. Age-related spatial reference and working memory deficits assessed in the water maze. Neurobiol Aging (1995) 16:149–60. 10.1016/0197-4580(94)00155-3 7777133

[26] Gieling ET Wehkamp W Willigenburg R Nordquist RE Ganderup N-C van der Staay FJ. Performance of conventional pigs and Göttingen miniature pigs in a spatial holeboard task: effects of the putative muscarinic cognition impairer biperiden. Behav Brain Funct (2013) 9:4–4. 10.1186/1744-9081-9-4 23305134 PMC3563551

[27] Gieling ET Park SY Nordquist RE van der Staay FJ Cognitive performance of low- and normal-birth-weight piglets in a spatial hole-board discrimination task. Pediatr Res (2012) 71:71–6. 10.1038/pr.2011.5 22289853

[28] Gieling ET Antonides S Fink-Gremmels J Ter Haar K Kuller WI Meijer E Chronic allopurinol treatment during the last trimester of pregnancy in sows: effects on low and normal birth weight offspring. PLoS One (2014) 9:e86396. 10.1371/journal.pone.0086396 24466072 PMC3899238

[29] Grimberg-Henrici CG Vermaak P Bolhuis JE Nordquist RE van der Staay FJ Effects of environmental enrichment on cognitive performance of pigs in a spatial holeboard discrimination task. Anim Cogn (Forthcoming). 10.1007/s10071-015-0932-7 PMC4751158 26520648

[30] Brevers D Bechara A Cleeremans A Noël X Iowa gamblingtask (IGT): twenty years after – gambling disorder and IGT. Front Psychol (2013) 4:14 10.3389/fpsyg.2013.00665 24137138 PMC3786255

[31] van den Bos R Koot S de Visser L. A rodent version of the Iowa gamblingtask: 7 years of progress. Front Psychol (2014) 5:6. 10.3389/fpsyg.2014.00203 24672498 PMC3957418

[32] Starcke K Brand M. Decision making under stress: a selective review. Neurosci Biobehav Rev (2012) 36:1228–48. 10.1016/j.neubiorev.2012.02.003 22342781

[33] de Vries M Holland RW Witteman CLM In the winning mood: affect in the Iowa gambling task. Judgm Decis Mak (2008) 3:42–50.

[34] Bechara A Damasio AR Damasio H Anderson SW Insensitivity to future consequences following damage to human prefrontal cortex. Cognition (1994) 50:7–15. 10.1016/0010-0277(94)90018-3 8039375

[35] Merlot E Mounier AM Prunier A. Endocrine response of gilts to various common stressors: a comparison of indicators and methods of analysis. Physiol Behav (2011) 102:259–65. 10.1016/j.physbeh.2010.11.009 21109031

[36] van der Staay FJ de Groot J van Reenen CG Hoving-Bolink AH Schuurman T Schmidt BH. Effects of butafosfan on salivary cortisol and behavioral response to social stress in piglets. J Vet Pharmacol Ther (2007) 30:410–6. 10.1111/j.1365-2885.2007.00884.x 17803732

[37] Velie BD Cassady JP Whisnant CS Endocrine response to acute stress in pigs with differing backtest scores. Livest Sci (2012) 145:140–4. 10.1016/j.livsci.2012.01.008

[38] van der Staay FJ Spatial working and reference memory of Brown Norway and WAG rats in a holeboard discrimination task. Neurobiol Learn Mem (1999) 71:113–25. 10.1006/nlme.1998.3860 9889077

[39] Gieling ET Musschenga MA Nordquist RE van der Staay FJ Juvenile pigs use simple geometric 2D shapes but not portrait photographs of conspecifics as visual discriminative stimuli. Appl Anim Behav Sci (2012) 142:142–53. 10.1016/j.applanim.2012.10.018

[40] Nawroth C Ebersbach M von Borell E. Juvenile domestic pigs (*Sus scrofa* domestica) use human-given cues in an object choice task. Anim Cogn (2014) 17:701–13. 10.1007/s10071-013-0702-3 24197275

[41] Anselme P. Does reward unpredictability reflect risk? Behav Brain Res (2015) 280:119–27. 10.1016/j.bbr.2014.12.003 25496783

[42] Murphy E Kraak L Nordquist RE van der Staay FJ Successive and conditional discrimination learning in pigs. Anim Cogn (2013) 16:883–93. 10.1007/s10071-013-0621-3 23525688

[43] de Jong IC Prelle IT van de Burgwal JA Lambooij E Korte SM Blokhuis HJ Effects of environmental enrichment on behavioral responses to novelty, learning, and memory, and the circadian rhythm in cortisol in growing pigs. Physiol Behav (2000) 68:571–8. 10.1016/S0031-9384(99)00212-7 10713299

[44] Hillmann E Schrader L Mayer C Gygax L. Effects of weight, temperature and behaviour on the circadian rhythm of salivary cortisol in growing pigs. Animal (2008) 2:405–9. 10.1017/S1751731107001279 22445043

[45] Ekkel ED Dieleman SJ Schouten WGP Portela A Cornélissen G Thielen MJM The circadian rhythm of cortisol in the saliva of young pigs. Physiol Behav (1996) 60:985–9. 10.1016/0031-9384(96)00107-2 8873280

[46] Ekkel ED Savenije B Schouten WGP Wiegant VM Thielen MJM. The effects of mixing on behavior and circadian parameters of salivary cortisol in pigs. Physiol Behav (1997) 62:181–4. 10.1016/S0031-9384(97)00037-1 9226360

[47] Escribano D Fuentes-Rubio M Cerón JJ. Validation of an automated chemiluminescent immunoassay for salivary cortisol measurements in pigs. J Vet Diagn Invest (2012) 24:918–23. 10.1177/1040638712455171 22914821

[48] Cook NJ Hayne SM Rioja-Lang FC Schaefer AL Gonyou HW The collection of multiple saliva samples from pigs and the effect on adrenocortical activity. Can J Anim Sci (2013) 93:329–33. 10.4141/CJAS2012-120

[49] Geverink NA Schouten WGP Gort G Wiegant VM Individual differences in behaviour, physiology and pathology in breeding gilts housed in groups or stalls. Appl Anim Behav Sci (2003) 81:29–41. 10.1016/S0168-1591(02)00253-8

[50] Sorrells AD Eicher SD Harris MJ Pajor EA Richert BT. Periparturient cortisol, acute phase cytokine, and acute phase protein profiles of gilts housed in groups or stalls during gestation. J Anim Sci (2007) 85:1750–7. 10.2527/jas.2007-0025 17468426

[51] Zhou Q Sun Q Wang G Zhou B Lu M Marchant-Forde JN Group housing during gestation affects the behaviour of sows and the physiological indices of offspring at weaning. Animal (2014) 8:1162–9. 10.1017/S1751731114001025 24801378

[52] Rault J-L Friends with benefits: social support and its relevance for farm animal welfare. Appl Anim Behav Sci (2012) 136:1–14. 10.1016/j.applanim.2011.10.002

[53] Ditzen B Heinrichs M. Psychobiology of social support: the social dimension of stress buffering. Restor Neurol Neurosci (2014) 32:149–62. 10.3233/RNN-139008 23603443

[54] Salehi B Cordero MI Sandi C. Learning under stress: the inverted-U-shape function revisited. Learn Mem (2010) 17:522–30. 10.1101/lm.1914110 20884754

[55] Sandi C Pinelo-Nava MT Stress and memory: behavioral effects and neurobiological mechanisms. Neural Plast (2007) 2007:78970 10.1155/2007/78970 18060012 PMC1950232

[56] Puppe B Ernst K Schön PC Manteuffel G Cognitive enrichment affects behavioural reactivity in domestic pigs. Appl Anim Behav Sci (2007) 105:75–86. 10.1016/j.applanim.2006.05.016

[57] Zebunke M Puppe B Langbein J. Effects of cognitive enrichment on behavioural and physiological reactions of pigs. Physiol Behav (2013) 118:70–9. 10.1016/j.physbeh.2013.05.005 23680428

[58] Siegford JM Rucker G Zanella AJ Effects of pre-weaning exposure to a maze on stress responses in pigs at weaning and on subsequent performance in spatial and fear-related tests. Appl Anim Behav Sci (2008) 110:189–202. 10.1016/j.applanim.2007.03.022

[59] Murphy E Nordquist RE van der Staay FJ Responses of conventional pigs and Göttingen miniature pigs in an active choice judgement bias task. Appl Anim Behav Sci (2013) 148:64–76. 10.1016/j.applanim.2013.07.011

